# Protein Variants Form a System of Networks: Microdiversity of IMP Metallo-Beta-Lactamases

**DOI:** 10.1371/journal.pone.0101813

**Published:** 2014-07-11

**Authors:** Michael Widmann, Jürgen Pleiss

**Affiliations:** Institute of Technical Biochemistry, University of Stuttgart, Stuttgart, Germany; University of South Florida College of Medicine, United States of America

## Abstract

Genome and metagenome sequencing projects support the view that only a tiny portion of the total protein microdiversity in the biosphere has been sequenced yet, while the vast majority of existing protein variants is still unknown. By using a network approach, the microdiversity of 42 metallo-β-lactamases of the IMP family was investigated. In the networks, the nodes are formed by the variants, while the edges correspond to single mutations between pairs of variants. The 42 variants were assigned to 7 separate networks. By analyzing the networks and their relationships, the structure of sequence space was studied and existing, but still unknown, functional variants were predicted. The largest network consists of 10 variants with IMP-1 in its center and includes two ubiquitous mutations, V67F and S262G. By relating the corresponding pairs of variants, the networks were integrated into a single system of networks. The largest network also included a quartet of variants: IMP-1, two single mutants, and the respective double mutant. The existence of quartets indicates that if two mutations resulted in functional enzymes, the double mutant may also be active and stable. Therefore, quartet construction from triplets was applied to predict 15 functional variants. Further functional mutants were predicted by applying the two ubiquitous mutations in all networks. In addition, since the networks are separated from each other by 10–15 mutations on average, it is expected that a subset of the theoretical intermediates are functional, and therefore are supposed to exist in the biosphere. Finally, the network analysis helps to distinguish between epistatic and additive effects of mutations; while the presence of correlated mutations indicates a strong interdependency between the respective positions, the mutations V67F and S262G are ubiquitous and therefore background independent.

## Introduction

Since 1951 when the protein sequence of insulin became available [1], the amount of available sequence information has been growing at an ever increasing rate [2]. Despite all efforts, it is assumed that the currently known sequences only represent a tiny portion of the largely unknown sequence space of the biosphere. Conservative estimations predict a sequence space size of around 10^37^ [3], while only around 2⋅10^8^ sequences are known today [2]. The ongoing discovery of new protein sequences, either being variants of already known proteins or completely new proteins, therefore can be interpreted as a stepwise exploration of the yet undiscovered sequence space, rather than a proof of an ongoing evolutionary mechanism. This question is especially relevant for proteins that transfer antimicrobial resistance. During the last 70 years, it has been observed that antibiotic resistances have emerged shortly after new antibiotics were introduced to the clinics. Is the occurrence of new resistance-mediating genes a proof of an ongoing evolutionary event, or did these new sequence emerge from a gene reservoir existing in the biosphere since long? The discovery of genes encoding resistances to β-lactam, tetracycline, and glycopeptide antibiotics in prehistoric soil samples without contact to synthetic antibiotics supports the latter possibility [4], [5]. Thus, evolution of resistance-mediating genes is ongoing since a long time, and it can be assumed that it has generated a large microdiversity of protein sequences. It is of major medical importance to establish a strategy to extract information about structure of the sequence space existing in the biosphere from the currently known few hundreds of variants.

The construction of phylogenetic trees from known protein sequences is a widely used method to analyze evolutionary relationships of proteins and to make predictions about ancestors of “modern” proteins [6]. It has become common practice to reconstruct and subsequently express ancestor proteins in order to create robust proteins that are expected to combine the biochemical properties of their more specialized offspring [7]–[9]. Tools for the alignment of multiple protein sequences and the construction of phylogenetic trees are numerous and have been steadily improved [10]–[12]. Under the assumption of an ongoing evolutionary process that is mostly described as a binary tree, generations of ancestor sequences are reconstructed providing ancestral, supposedly extinct links between the different protein variants that exist today. Although phylogenetic trees are intuitive to biologists, straightforward to analyze, and widely used, they often fail to adequately represent the relationships between proteins and might lead to wrong biological conclusions since only a tiny portion of the sequence space of contemporary proteins is known yet. This includes the methods of ancestor reconstruction and the assumption of a steadily expanding protein universe [6].

As an alternative to describing homologous sequences by phylogenetic trees, protein sequences can be viewed as a network [13]. The two major advantages of a network representation over a phylogenetic tree are that it lacks any assumption about ancestral sequences and that it directly uses the observed sequence similarities as the metric of the network. In this work, we apply a network approach to analyze evolutionary relationships between closely related variants of metallo-β-lactamases (MBLs) [14] [15]. MBLs have caused major concerns due to their efficient inactivation of most β-lactams [16]–[18] and the absence of clinically useful inhibitors [16], [19]. A better understanding of the sequence-function relationship of these enzymes and the mechanism of how mutations are acquired under the selective pressure of antibiotics and MBL inhibitors is crucial for the design of novel antibacterial drugs with long-term efficacy [20]. New MBL variants are discovered at a steady pace, making more and more information about protein variants available. In order to analyze MBLs, protein databases have been established and naming schemes have been developed [21] to allow for the identification of unique variants and mutated positions. The IMP subfamily [16], [18] constitutes the clinically most significant MBLs. More than 30 IMP variants have been found in clinical isolates globally. The number of mutations between the different variants ranges between 1 and 100. The IMP family of MBLs was chosen as a model system, because many closely related variants have been described and most of the variants were biochemically characterized and described in literature due to their impact on the modern healthcare system [16], [20]. A database on MBLs and a numbering scheme for amino acid positions was previously established, which allows for an unambiguous comparison of variants [21], [22]. Most important, a naming scheme for IMP variants exist which assigns a unique number to each variant in the approximately temporal order of its first occurrence [15].

## Methods

### IMP sequences

The Metallo-β-Lactamase Engineering Database (MBLED) served as the source of the protein sequences of the IMP family. The MBLED is publicly accessible at http://www.LacED.uni-stuttgart.de/and consists of 517 protein entries assigned to three subclasses B1, B2, and B3. The homologous family of IMP metallo-β-lactamases belongs to subclass B1 and consists of 81 protein sequences of which 42 belong to non-identical protein sequences (Information S1, see Table S1) which were included in this study.

A multisequence alignment of the 42 sequences was generated using ClustalW [12] with a gap opening and a gap extension penalty of 10 and 0.2, respectively, and using a GONNET score matrix. A 42×42 distance matrix was derived by counting the number of amino acid differences (including deletions) between each pair of sequences. The resulting distance matrix was used for constructing sequence networks as well as a neighbor-joining tree using the PHYLIP package [23].

### Network construction

In the previously published numbering scheme [21] for the IMP family, IMP-1 was chosen as the reference sequence. Each IMP sequence was compared to the reference sequence IMP-1, and the amino acid differences relative to IMP-1 were used to construct a mutation profile. By comparing the mutation profiles of all IMP sequences, proteins which share mutations are easily identified (Table S1 in Information S1). All pairs of protein sequences that differed by a single mutation were linked. Sequence pairs that differed by two mutations were linked by inserting an unknown sequence annotated by a question mark. In total, 7 networks were constructed with at least 3 IMP sequences linked by single or double mutations In each network, the IMP sequence with the smallest number of mutations in respect to IMP-1 was identified, which served as the core of the respective network. By connecting IMP-1 and the other core sequences of each network, all networks were integrated into a single system of networks.

## Results

### 1. Representing distances by phylogenetic trees and networks

The sequences of 42 IMP metallo-β-lactamases were aligned and a 42×42 distance matrix was established by counting the number of different amino acids in each pair of sequences. Most of the sequences were tightly connected. Among the 42 IMP sequences, 23 sequence pairs were found that differed by only one amino acid. 36 sequences had an assigned IMP number, 6 sequences from the GenBank belonged to the IMP family, but had not yet an assigned IMP number (Table S1 in Information S1) [22].

**Figure 1:**
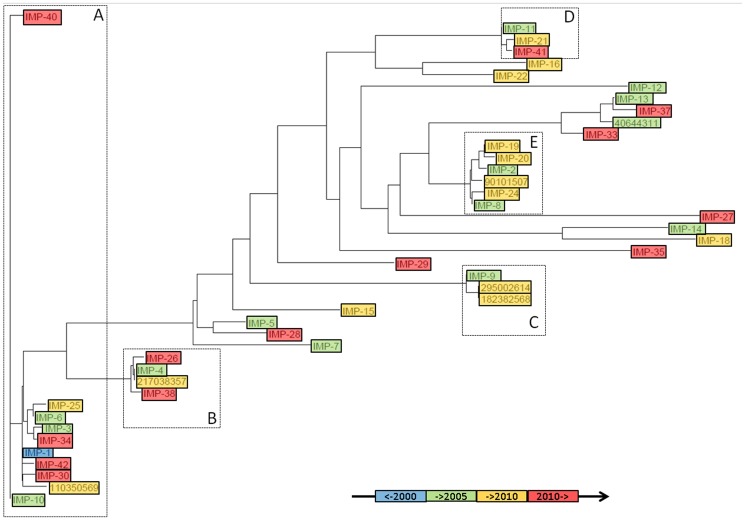
Phylogenetic tree of the IMP family of MBLs, colored by time of discovery (blue: before 2000, green: 2000-2005, yellow: 2005–2010, red: after 2010).

**Figure 2:**
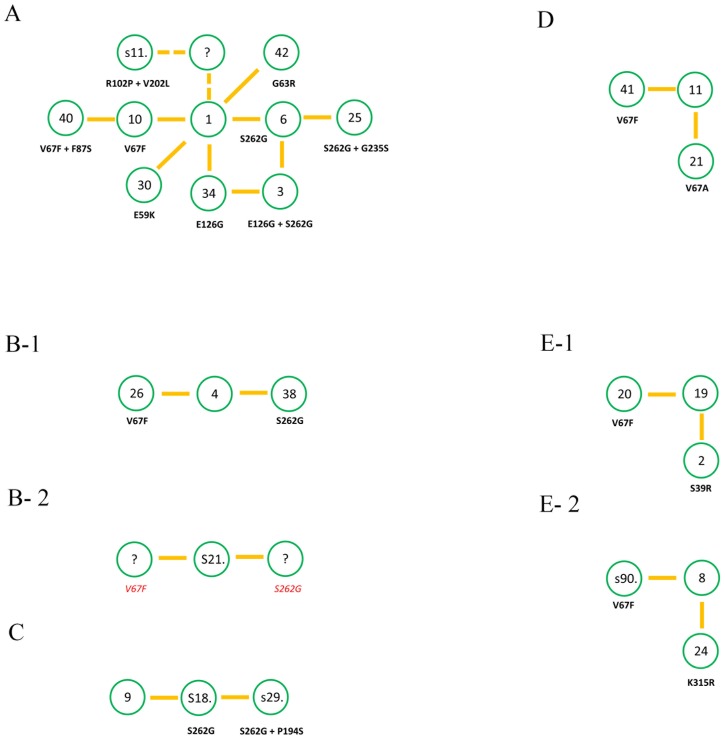
Networks of the five main branches A-E. The nodes are labeled by their IMP number (e.g. IMP-1 is labeled by “1”). If no IMP number has been assigned yet, the abbreviated GenBank ID is given (e.g. the GenBank entry 110350569 is labeled by “s11.”). Unknown variants are labeled by “?”.

A phylogenetic tree revealed five branches with at least three closely related variants (Figure 1). The largest branch A consists of 10 IMP variants, IMP-1, IMP-3, IMP-6, IMP-10, IMP-25, IMP-30, IMP-34, IMP-40, IMP-42, and the not yet assigned sequence s110 (GenBank entry 110350569). Branch B consists of 4 variants, IMP-4, IMP-26, IMP-38, and s217 (GenBank entry 217038357), branch C of 3 variants, IMP-9, s182 (GenBank entry 182382568), and s295 (GenBank entry 295002614), branch D of 3 variants, IMP-11, IMP-21, and IMP-41, and branch E of 6 variants, IMP-2, IMP-8, IMP-19, IMP-20, IMP-24, and s901 (GenBank entry 90101507). 16 IMP variants belonged to branches with less than three variants. From each of the five main branches, networks were constructed by connecting all variants that differ by a single or a double mutation (Figure 2). For the 10 variants of network A, the number of neighbors of each variant varies between 6 (IMP-1) and 1 (IMP-25, IMP-40, and IMP-42). Sequence s110 differed by two mutations (R102P and V202L) from its closest neighbor IMP-1. We assume that 2 yet unknown IMP variants exist, which differ from IMP-1 by one mutation (R102P and V202L, respectively) and form a link between IMP-1 and s110. This assumption is supported by the observation that a quartet of variants (IMP-1, IMP-3, IMP-6, and IMP34) was found in network A, where IMP-3 differs from IMP-1 by two mutations (E126G and S262G), while IMP-34 (E126G) and IMP-6 (S262G) differ from IMP-1 by a single mutation.

For closely related variants which differ by only a few mutations, the representation of the distance matrix by a network is preferable to its representation by a tree, because the distances between variants can be unequivocally derived by measuring the nearest path through the network. In contrast, distances represented by any phylogenetic tree are inconsistent with the distance matrix of a set of variants that are members of closed paths. This is most pronounced for the quartet IMP-1, IMP-3, IMP-6, and IMP34 (Figure S1 in Information S1). The fact that four pairs of variants differ by one mutation (IMP-1/IMP-6, IMP-6/IMP-3, IMP-3/IMP-34, IMP-34/IMP-1) and two pairs differ by two mutations (IMP-1/IMP-3, IMP-6/IMP-34) cannot be consistently represented by any binary tree (Figures S2, S3 in Information S1). The inadequacy of phylogenetic trees constructed from closely related sequences is confirmed by comparing a tree and a network derived from the distance matrix of network A. The distance between IMP-3 and IMP-6 in the phylogenetic tree is nearly twice as large as the distance between IMP-3 and IMP-34 (Figure 1), while the distance between each IMP pair (1 mutation) should identical (Figure 2).

### 2. System of coupled networks

**Figure 3:**
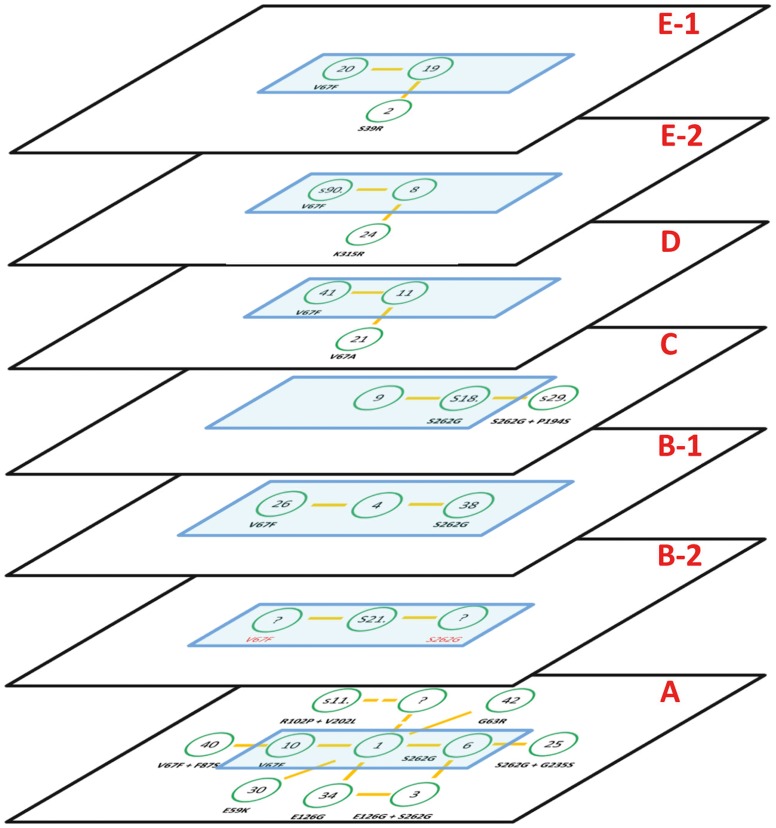
System of 7 networks. Each network is arranged in a separate plane. The core variants (IMP-1, s217, IMP-4, IMP-9, IMP-11, IMP-8, IMP-19) and the variants derived thereof by the mutations V67F or S262G are marked (blue rectangle) and positioned on top of each other.

For each of the initial networks, a core sequence was determined; IMP-1 in network A as well as the variant with closest distance to IMP-1 in network B to E (IMP-4, IMP-9, IMP-11, and IMP-19, respectively). While the maximum distance between two variants in the networks ranges between 2 (networks B, C, D) and 4 mutations (network A), the distance between the core sequences of the five networks ranges between 10 (network A - network B) and 37 mutations (network A - network D). Thus, at a first glance, the networks seem to be unrelated. However, each pair of networks has at least one mutation in common. Mutation V67F was found in networks A (IMP1/IMP-10), B (IMP-4/IMP-26), D (IMP-11/IMP41)), and twice in network E (IMP-19/IMP-20 and IMP-8/s901). Mutation S262G was found in networks A (IMP-1/IMP-6), B (IMP-4/IMP-38), and C (IMP-9/s182). To relate the five networks, the planes representing each network were oriented in parallel, and the core sequences and the pairs of variants with identical mutations were positioned on top of each other (Figure 3). Since the mutation V67F was found twice in network E, this network was separated into two sub-networks E-1 and E-2 with a distance of one mutation, and IMP-19 and IMP-8, respectively, as core sequences.

Network A and B share two mutations, V67F and S262G, which connect two IMP triplets; V67F links IMP-1 and IMP-10 in network A, and IMP-4 and IMP-26 in network B. S262G links IMP-1 and IMP-6 in network A, and IMP-4 and IMP-38 in network B. Thus, IMP-1 in network A corresponds to IMP-4 in network B, IMP-10 to IMP-26, and IMP-6 to IMP38, and all three pairs differ by the same tenfold mutation (S39P, N77D, R132Q, T170K, S174G, V201L, I241L, K252I, V266A, P320L) (Table S2 in Information S1). We assume that IMP-1 and IMP-4 are connected by a path of at least ten subsequent single mutations resulting in functional variants. Interestingly, one mutation along this path is already known, S39P which links s217 and IMP-4. Thus, network B was separated into two sub-networks B-1 and B-2 with IMP-4 and s217 as core sequences, differing from IMP-1 by 10 and 9 mutations, respectively. Since both mutations, V67F and S262G are found in network B-1, we would assume that both mutations of s217 in network B-2 would also result in active proteins, as indicated by a question mark in Figure 2.

Similarly, the other networks were aligned. Network C contains mutation S262G, linking IMP-9 and s182. Thus, IMP-9 was related to IMP-1, s182 to IMP- 6. Network D contains mutation V67F, linking IMP-11 and IMP-41. Thus, IMP-11 was related to IMP-1, IMP-41 to IMP- 10. Networks E-1 and E-2 contain mutation V67F, linking IMP-8 and s901, and IMP-19 and IMP-20, respectively. Thus, IMP-8 and IMP-19 were related to IMP-1, s901 and IMP-20 to IMP-10.

Apart from these two mutations that are shared by most of the five networks, all other mutations are yet unique to each network.

### 3. Prediction of missing links

The networks A to E represent the sequence space of the IMP sequences that is known today. The fact that these IMP variants exist as networks indicates that single mutations of stable and catalytically active proteins have a high chance of resulting in stable and active proteins. This assumption is strongly supported by the existence of highly connected variants, such as the quartet IMP-1, IMP-6, IMP-34 and IMP-3 in network A.

The sequence space near to the existing networks was analyzed to predict variants that are closely related to known variants. These unknown variants are expected to be stable and catalytically active enzymes, and therefore might exist in the biosphere without having been discovered yet. We suggest three strategies to construct these missing links.

1. Quartet construction. The existence of missing links is most probable for cases where three variants 1, 2, and 3 in the network are linked by single mutations between variant 1 and 2 and between variant 2 and 3, and two mutations between variant 1 and 3, such as the triplet IMP-40 – IMP-10 - IMP-1 with the mutation F87S linking IMP-40 and IMP-10 and mutation V67F linking IMP-10 and IMP-1 (Figure 2). The missing mutation F87S of IMP-1 has a high probability of being stable and active. There are 15 triplets in the networks where an additional variant would result in a quartet of variants linked by single mutations (Table S3 in Information S1). This quartet construction could be further used in an iterative process to predict new variants by combining existing and previously predicted variants, leading to a rapidly increasing number of predicted variants. Quartets of variants were also constructed from a pair of variants that differ by two mutations, such as IMP-1 and s110 (R102P and V202L). There are two variants that link IMP-1 to s110 resulting from the single mutations R102P and V202L. Because both variants differ from two functional enzymes by only one mutation each, there is a high probability that they are also function (Table S4 in Information S1).
2. Identification of mutation-prone positions. Not all positions are equally probable of being mutated successfully. The most frequently mutated positions are 67 and 262. Mutation V67F occurred 5 times in different backgrounds (in networks A, B-1, D, E-1, and E-2), mutation S262G 3 times (in networks A, B-1, and C). In networks B-1 and A, both mutations were observed (Figure 2). In networks E-1, E-2 and D, mutation V67F was observed, but not mutation S262G. Network C contained the mutation S262G, but not mutation V67F. In network B-2, both mutations were absent. Because these two mutations seem to be ubiquitous, we would expect them to result in active variants independent of the background. Thus, we predict 2 variants with mutation V67F in networks B1 and C and 4 variants with mutation S262G in networks B1, C, E-1, and E-2 to be functional (Table S5 in Information S1).
3. Pathways between networks. It is expected that the empty sequence space between the layers also contain stable and active variants that have not been discovered yet. In contrast to quartet construction and the identification of mutation-prone positions, there are multiple hypothetical pathways between the layers. Therefore, only the shortest pathways requiring a minimal number of exchanges can be constructed. Using this approach, there are three possible pathways, connecting network A and B-1: ten mutations connecting the pairs IMP-1/IMP-4, IMP-10/IMP-26, and IMP-6/IMP-38. However, it cannot be predicted whether the 10 mutation have to occur in a specific order, or whether all combinations result in active enzymes.

## Discussion

Since the advent of the first metallo-β-lactamase of the IMP type in 1994 [24], the number of known sequences has steadily increased. It has been assumed that new mutations occurred due to selective pressure by widespread use of antibiotics [18], [19]. Since new variants were numbered in the order of their occurrence, the IMP-numbering scheme provides an approximate timeline for the discovery of new variants. IMP-1 was in detected in 1994, IMP-2 to IMP-26 between 1998 and 2010, IMP-27 to IMP-44 since 2010. If the new variants were a result from an ongoing evolution, we would expect that the sequence space covered by the IMP sequences was continually expanding. As a consequence, the IMP number of a variant would relate to its location in the tree, with the most recent variants having the longest distance from the root, and the older variants being closer to the root. However, the date of discovery and the subsequent numbering is no indication of a variant's position in relation to the root (Figure 1). This is demonstrated for the variants IMP-40 and IMP-41. Both sequences were published on the same day (10.10.2012), but their position in IMP sequence space differs considerably. IMP-40 is only two mutations away from the first variant IMP-1 discovered in 1994, while IMP-41 differs from IMP-1 by 38 mutations. Network A consists of 10 variants which differ from IMP-1 by only a few mutations. While some variants were discovered soon after the discovery of IMP-1, such as IMP-3 (1998) and IMP-6 (2000), other variants in network A have only been recently discovered, such as IMP-40 and IMP-42. The same observation applies to the other networks. Therefore, the sequence space of MBLs seems to be gradually populated by the discovery of new sequences in a random order. It is expected that more and more variants will be discovered, filling the empty sequence space inside and between the IMP networks. This also implies that the first IMP sequences that have been detected 20 years ago are not necessarily ancestors of the IMP family. Instead, we seem to randomly pick and sequence variants from a protein pool that is pre-existing in the biosphere. A subset of this pool is continuously screened in the clinics for active and stable variants that confer antibiotic resistance.

In 1970, J. Maynard Smith introduced the concept of protein sequence space and suggested a model of protein evolution in a network of functional proteins [13]. Under the assumption that “functional proteins must form a continuous network which can be traversed by unit mutational steps without passing through nonfunctional intermediates”, the gaps in the sequence space of today's proteins are a consequence of the limited amount of sequence information that is known today. Talking about the size of the sequence space, a distinction has to be made between the theoretical sequence space (the part of sequence space that can be reached by a certain number of mutations) and the functional or viable sequence space (sequences with a certain number of mutations that result in active proteins and therefore probably exist in the biosphere). Assuming a total of 10^30^ cells in the biosphere, each expressing 10^4^ different proteins [25] with 10^3^ bases, the size of the sequence space of the biosphere can be estimated to 10^37^ bases [3]. Therefore, the viable sequence space is about 26 orders of magnitude larger than the 150•10^9^ bases that have been sequenced today [2]. Because we therefore expect to see only an infinitesimal part of the viable sequence space, it is not surprising that there are large gaps in the IMP networks. Thus, the five networks of 26 IMP variants and the additional 16 scattered IMP variants are expected to be embedded in a highly connected sequence network of 10^27^–10^28^ variants that exist in the biosphere in this region of sequence space (about 26 orders of magnitude larger than the number of known proteins today). This assumption is in accordance with the observation that MBLs possess a robust fold which tolerates second-sphere mutations and therefore allows for a large number of functional variants [26]. However, not all predicted mutation profiles are expected to result in viable enzymes. The absence of an expected mutation profile would be a strong indication of spatial restraints which might be understood by analyzing the currently available 3D structures.

The maximum distance between the IMP variants in the coupled network is 40 mutations. Thus, the theoretical sequence space is 20^40^ = 10^52^, 25 orders of magnitude larger than the estimated viable sequence space of IMP variants. Thus, although the expected number of IMP variants existing in the biosphere is very large (10^27^–10^28^), they occupy only an infinitesimal part of the theoretical sequence space. Given this situation, how is it possible to predict unknown viable sequences using the available information from the known sequence space? We suggest that the construction of networks is a promising strategy to predict existing but not-yet-discovered sequences in the nearly infinite sea of theoretical variants.

But what is the advantage of a network-based analysis as compared to a tree-based analysis? In general, the relation between protein sequences and their assignment to protein families is based on the construction of a binary tree. Tree reconstruction methods such as neighbour-joining, maximum likelihood, or maximum parsimony [27], [28] assume the existence of ancestor sequences and an additivity of distances between extinct ancestors and the existing sequences. If multiple trees of similar probability are found, a consensus tree is constructed which might contain none of them [29]. In addition, there might be more than one path to get from an ancestor to a contemporary protein. Therefore, any tree representation will consistently fail to represent a protein family formed by a densely connected network of single and double mutants. The distance matrix of a triplet of sequences consisting of a sequence and two single mutants thereof (Figure S1 in Information S1), where the distance between the two mutants is 2, cannot be adequately represented in a binary tree with the three sequences as leafs and assuming additivity of distances. Similarly, a quartet of sequences consisting of a sequence, two single mutants thereof, and the respective double mutant (Figure S2 in Information S1) can easily be represented in a network with the shape of a square of side length of 1 and diagonal distances of 2 mutations (Figure S3 in Information S1). However, this distance matrix cannot be represented in any binary tree assuming additivity of distances. Therefore, phylogenetic trees fail to assess highly connected networks of closely related variants.

In contrast, the representation of variants as networks is a valuable tool to analyze relationships between closely related variants and to predict variants that have not yet been sequenced, but which are expected to exist in the biosphere. These predictions can be made on two different levels of the network.

1. On a local network level, this is demonstrated for IMP-34, linking IMP-1, IMP-6, and the double mutant IMP-3, resulting in a quartet of sequences. While IMP-1, IMP-3, and IMP-6 were already known, it took 11 years before IMP-34 was also discovered. In addition, we expect to find for each triplet of point mutants (Figure S1 in Information S1) a fourth, not-yet-discovered variant that results in a square arrangement of the four variants (Figures S2, S3 in Information S1). Thus, from a single network of four variants of known sequence, three variants can be predicted that are expected to be functional and to exist in the biosphere (Figure S4 in Information S1). Under the assumption that these predicted sequences exist as functional and active proteins, in further rounds of construction additional variants can be predicted from each of the new triplets (Figure S5 in Information S1).
2. On the level of coupled networks, IMP-41 is an example of predicting new functional variants. IMP-41 was discovered in 2012, but would have been predicted before, because it is derived from IMP-11 (detected before 2005) by the ubiquitous mutation V67F (Figure 2). Likewise, further V67F and S262G mutants are expected to exist in all of the remaining networks.

A highly connected network was constructed consisting of variants which have a high probability to fold, to be functional, and therefore to exist in the biosphere. The large number of possible variants prevents purely random approaches to predict new functional variants. Coupled networks of known variants provide a guideline to predict functional, but yet unknown variants. This includes variants that are distant in sequence space in regard to the total number of mutations, but share mutations that can be identified by systematically comparing the networks.

A different approach for the prediction of functional variants is the detection of correlated mutations in a given protein family which can be seen as an extreme case of background dependence. In contrast, the network approach is better suited to identify functional mutations that are background independent, as each layer in the network represents a different set of background mutations. The complementarity of both methods is demonstrated analyzing the IMP family for correlated mutations using COMULATOR [30], resulting in three sets of correlated mutations 37-79-97, 49-68-198, and 208-319 (IMP numbering). These positions are part of the background mutations that constitute the different networks, and therefore were not identified by our network approach. In contrast, the frequent mutations V67F and S262G were present in many different networks and therefore are supposed to be background-independent.

It was shown that the construction of coupled networks is a valuable tool to gain new insights into the sequence space of a given protein family. It allows for the systematic analysis and prediction of missing sequences on a local level as well as on a global family level. While the well characterized sequence space of IMP variants is ideally suited to take advantage of a network representation, this approach is generic. It could be applied to any protein family to predict new variants and missing links by navigating in today's known sequence space.

## Supporting Information

Information S1 **Table S1**. Protein names, gene identifers and mutation profiles of all IMP sequence used in the construction of phylogenetic trees and protein networks. Gene identifiers were taken from (http://www.lahey.org) where available. **Table S2**. Mutations shared by all variants of the respective network in comparison to the reference sequence IMP-1. **Table S3**. Triplets of IMP variants with predicted missing variant to complement the triplet to a quartet. **Table S4**. Pairs of IMP variants which differ by 2 mutations with no know intermediary variants. Intermediary variants for possible pathways were predicted. **Table S5**. Predicted S262G and V67F variants. **Figure S1**. The distance matrix and a network representation of a triplet of sequences consisting of a wild type and two single mutants thereof (mutant 1 and mutant 2). **Figure S2**. The distance matrix of a quartet of sequences consisting of a wild type, two single mutants thereof (mutants1 and 2), and the respective double mutant. **Figure S3**. Left: Network representation of a quartet of sequences consisting of a wild type, two single mutants thereof (mutants1 and 2), and the respective double mutant (derived from the distance matrix in Figure S2). Right: the four sequences cannot be represented by a binary tree assuming additivity of distances. **Figure S4**. From a network of four variants of known sequence (black), three variants (red) can be predicted that are expected to be functional and to exist in the biosphere. **Figure S5**. From the network constructed in Figure S4, four further mutants (green) can be predicted that are expected to be functional and to exist in the biosphere.
(PDF)
