# Supplementary material for: Protein Variants Form a System of Networks: Microdiversity of IMP Metallo-Beta-Lactamases
Source: PLoS One. 2014 Jul 11;9(7):e101813. doi: 10.1371/journal.pone.0101813 (PMC4094381; doi:10.1371/journal.pone.0101813)
Supplement: Information S1 — Table S1. Protein names, gene identifers and mutation profiles of all IMP sequence used in the construction of phylogenetic trees and protein networks. Gene identifiers were taken from (http://www.lahey.org) where available. Table S2. Mutations shared by all variants of the respective network in comparison to the reference sequence IMP-1. Table S3. Triplets of IMP variants with predicted missing variant to complement the triplet to a quartet. Table S4. Pairs of IMP variants which differ by 2 mutations with no know intermediary variants. Intermediary variants for possible pathways were predicted. Table S5. Predicted S262G and V67F variants. Figure S1. The distance matrix and a network representation of a triplet of sequences consisting of a wild type and two single mutants thereof (mutant 1 and mutant 2). Figure S2. The distance matrix of a quartet of sequences consisting of a wild type, two single mutants thereof (mutants1 and 2), and the respective double mutant. Figure S3. Left: Network representation of a quartet of sequences consisting of a wild type, two single mutants thereof (mutants1 and 2), and the respective double mutant (derived from the distance matrix in Figure S2). Right: the four sequences cannot be represented by a binary tree assuming additivity of distances. Figure S4. From a network of four variants of known sequence (black), three variants (red) can be predicted that are expected to be functional and to exist in the biosphere. Figure S5. From the network constructed in Figure S4, four further mutants (green) can be predicted that are expected to be functional and to exist in the biosphere. (PDF) [file pone.0101813.s001.pdf]

Supporting information

Protein variants form a system of networks: microdiversity of IMP metallo-β-lactamases

Michael Widmann, Jürgen Pleiss*

Institute of Technical Biochemistry, University of Stuttgart, Allmandring 31, 70569 Stuttgart, Germany

* Corresponding author:

Jürgen Pleiss
Institute of Technical Biochemistry
University of Stuttgart
Allmandring 31
70569 Stuttgart, Germany
E-mail: Juergen.Pleiss@itb.uni-stuttgart.de

Tables

Table S1. Protein names, gene identifers and mutation profiles of all IMP sequence used in the construction of phylogenetic trees and protein networks. Gene identifiers were taken from (http://www.lahey.org) where available.

Name	Identifier	Mutation-Profile
IMP-1	S71932	
IMP-2	AJ243491	S20K, S23F, F25L, F26C, I27V, F28C, L29F, F30L, A34T, T35A, A37G, E38A, S39R, D49E, P68S, A78T, E79D, K90T, T97N, S112T, R132Q, T170K, N177S, P198Q, R208K, I223V, Y227D, I241L, L250I, K252M, G254bV, P261S, V266I, L302R, L304W, K319Q
IMP-3	AB010417	E126G, S262G
IMP-4	AF244145	S39P, N77D, R132Q, T170K, S174G, V201L, I241L, K252I, V266A, P320L
IMP-5	AF290912	S23F, I27M, A34T, T35A, A78T, R132Q, T170K, V176A, N177S, N183K, R208N, K215R, I216V, I223V, I241V, K252M, L302R
IMP-6	AB040994	S262G
IMP-7	AF318077	S20K, I27M, T35A, A36S, A37G, S39A, A78T, E79D, R132Q, T170K, V176A, N177S, N183K, V187I, R208H, K215R, I216V, I223V, I241L, K252V, L302R, P317L
IMP-8	AF322577	S20K, S23F, F25L, F26C, I27V, F28C, L29F, F30L, A34T, T35A, A37G, E38A, S39A, D49E, P68S, A78T, E79D, K90T, T97N, S112T, R132Q, T170K, N177S, P198Q, R208K, I223V, Y227D, I241L, L250I, K252M, P261S, V266I, L302R, L304W, K319Q
IMP-9	AY033653	S23F, I27M, A34T, T35A, A37G, V67I, A78T, E79D, K94N, T97N, K108R, R132Q, T170K, N171Y, N177S, N183K, T197A, R208N, K215R, I216V, I223V, I241L, K252M, G254bS, E265D, V266I, A297S, L304W, A307T, L311F, P317S, S318T, K319T, P320A, S321H, N322-
IMP-10	AB074433	V67F
IMP-11	AB074436	S20K, S23F, F25L, F26C, A34T, T35A, A37G, E38A, D49E, P68S, A78T, E79D, T97N, R132Q, E140V, T170K, N177S, P198Q, E207K, R208N, I223V, G235D, I241V, K246H, K249E, L250K, K252I, K255N, E265D, V266I, L304W, P317S, S318N, K319T, P320V, S321H, N322-
IMP-12	AJ420864	S20K, S23F, F25L, F26C, C31L, A34T, T35A, A36S, A37G, S39V, D49E, V54L, N62S, P68T, A78N, E79D, A89N, T97A, E101G, Y104F, K108T, I113V, R132Q, D149N, N177S, V187I, P198Q, R208N, I223V, Y227D, G235D, I241L, E242K, L250I, K252M, P261S, S262G, V266I, D296N, L304W, N312K, S318L, K319L
IMP-13	AJ550807	S20K, S23F, F25L, F26C, I27V, F28C, L29F, A34T, T35A, A37G, E38A, S39A, D49E, Y53F, P68T, A78T, E79D, K90T, T97N, K108E, S112T, R132Q, D149S, T170K, N171Y, G175E, N177S, P198Q, V201L, R208S, Y227H, I241L, L250I, K252M, P261S, V266K, L300M, L302R, L304W, V308L, N312K, P317T, K319S
IMP-14	AY553332	S20K, S23F, F25L, F26C, I27V, L29F, S32N, T35V, A37E, D49E, N62K, G65S, P68T, N77K, A78N, E79D, F87I, T97N, S115T, S119G, G123A, R132Q, G150N, T170K, N171H, S174N, N177S, W179S, V181I, P198Q, R208K, I223V, Y227D, N233Y, I241L, L250I, K252M, P261S, E265D, V266I, A297V, L302R, L304W, P317S, K319Q, N322D
IMP-15	AY553333	S20N, I27M, L29M, A34T, T35A, A37G, A78T, R132Q, T170K, V176G, N177S, K182N, R208N, K215R, I216V, I223V, I241L, L250I, K252M, P261S, V266T, D296N, L304W, N312K, K319L
IMP-16	AJ584652	S20K, S23F, F25L, F26C, A34T, T35A, A37G, D49E, E50D, P68T, V74F, A78T, E79D, T88A, T97N, T122S, R132Q, D149N, T170K, N177S, V181L, V187I, P198Q, R208K, I223V, G235D, I241V, K246H, K249E, L250I, K252M, K254R, K255N, E265D, L304W, N312K, K319Q
IMP-18	AY780674	S20K, S23F, F25L, F26C, I27V, L29F, F30L, S32N, T35A, A37D, E38D, D49E, E50K, N62K, P68T, N77K, A78N, E79D, F87I, T97N, V100I, R102H, K108R, S115T, S119G, G123A, R132Q, P135S, G150N, N177S, W179S, V181I, P198Q,

		R208K, I223V, Y227D, I241L, L250I, K252M, P261S, V266I, D296N, K301Q, L302R, L304W, S318L, K319Q, N322S
IMP-19	EF118171	S20K, S23F, F25L, F26C, I27V, F28C, L29F, F30L, A34T, T35A, A37G, E38A, S39A, D49E, P68S, A78T, E79D, K90T, T97N, S112T, R132Q, T170K, N177S, P198Q, R208K, I223V, Y227D, I241L, L250I, K252M, G254bV, P261S, V266I, L302R, L304W, K319Q
IMP-20	AB196988	S20K, S23F, F25L, F26C, I27V, F28C, L29F, F30L, A34T, T35A, A37G, E38A, S39A, D49E, V67F, P68S, A78T, E79D, K90T, T97N, S112T, R132Q, T170K, N177S, P198Q, R208K, I223V, Y227D, I241L, L250I, K252M, G254bV, P261S, V266I, L302R, L304W, K319Q
IMP-21	AB204557	S20K, S23F, F25L, F26C, A34T, T35A, A37G, E38A, D49E, V67A, P68S, A78T, E79D, T97N, R132Q, E140V, T170K, N177S, P198Q, E207K, R208N, I223V, G235D, I241V, K246H, K249E, L250K, K252I, K255N, E265D, V266I, L304W, P317S, S318N, K319T, P320V, S321H, N322-
IMP-22	DQ361087	S20K, S23F, F25L, F26C, I27V, A34T, T35A, A37G, D49E, P68S, V74I, A78T, E79D, R132Q, E144D, K148Q, D149N, T170K, N177S, P198Q, R208K, I223V, G235D, I241V, E242V, K246H, K249E, L250I, K252M, K254R, K255N, E265D, V266I, L304W, N312K, K319E
IMP-24	EF192154	S20K, S23F, F25L, F26C, I27V, F28C, L29F, F30L, A34T, T35A, A37G, E38A, S39A, D49E, P68S, A78T, E79D, K90T, T97N, S112T, R132Q, T170K, N177S, P198Q, R208K, I223V, Y227D, I241L, L250I, K252M, P261S, V266I, L302R, L304W, K315R, K319Q
IMP-25	EU541448	G235S, S262G
IMP-26	GU045307	S39P, V67F, N77D, R132Q, T170K, S174G, V201L, I241L, K252I, V266A, P320L
IMP-27	JF894248	S20K, S23F, F25L, F26C, I27V, L29V, A34T, T35V, A37G, S39T, D42N, K44R, I45V, D49E, F58Y, N62K, P68T, V76I, N77G, E79D, T97N, S112T, I113V, R132Q, T170K, S174D, N177S, V181A, N183D, P198Q, R208K, K215E, I223V, Y227H, I241L, K246E, L250I, K252M, S253E, P261S, S262G, V266T, S298T, L299H, L302R, L304W, N312K, P317T, S318L, K319Q
IMP-28	JQ407409	S23F,I27M,A34T,T35A,K47R,A78T,E101G,R132Q,T170K,S174G,N177S,R208N,K215R,I216V,I223V,I241L,K252M,L302R,Q306H
IMP-29	HQ438058	S23F,F26L,A34T,T35A,A78T,R132Q,D149G,T170K,N177S,N183K,R208N,K215R,I216V,I223V,G235D,I241V,K246H,K249E,L250I,K252M,K254R,K255N,E265D,V266I,D296N,L304W,N312K,K319Q
IMP-30	DQ522237	E59K
IMP-33	JN848782	S20K,S23F,F25L,F26C,I27V,F28C,L29F,A34T,T35A,A37G,E38S,D49E,Y53F,P68T,A78T,E79D,K90T,T97N,S112T,R132Q,D149S,T170K,N171Y,G175E,N177S,P198Q,V201L,R208S,I223V,Y227H,I241L,L250I,K252M,P261S,V266K,L302R,L304W,V308L,N312K,P317T,K319S
IMP-34	AB715422	E126G
IMP-35	JF816544	S20K,L22I,S23F,F25L,I27V,A34T,T35A,A37G,L43I,G51D,V61D,V67I,P68T,A78T,E79D,L82I,T97R,E101G,T122A,R132Q,E140K,D149N,K150aN,V167A,T170E,N177S,N183H,P198Q,R208K,Y227D,N233Y,I241L,K249E,L250T,K252M,K255N,P261S,V266I,D296G,L302R,L304W,N312K,K319Q,S321N
IMP-37	JX131372	S20K,S23F,F25L,F26C,I27V,F28C,L29F,A34T,T35A,A37G,E38A,S39A,D49E,Y53F,P68T,A78T,E79D,K90T,T97N,K108E,S112T,R132Q,D149S,T170K,N171Y,G175E,N177S,P198Q,V201L,R208S,Y227H,I241L,L250I,K252M,P261S,V266K,L300M,L302R,L304W,V308L,N312K,P317T,K319S,P320Q,N322T
IMP-38	HQ875573	S39P,N77D,R132Q,T170K,S174G,V201L,I241L,K252I,S262G,V266A,P320L
IMP-40	AB753457	V67F,F87S
IMP-41	AB753458	S20K,S23F,F25L,F26C,A34T,T35A,A37G,E38A,D49E,V67F,P68S,A78T,E79D,T97N,R132Q,E140V,T170K,N177S,P198Q,E207K,R208N,I223V,G235D,I241V,K246H,K249E,L250K,K252I,K255N,E265D,V266I,L304W,P317S,S318N,K319T,P320V,S321H,N322-
IMP-42	AB753456	G63R
90101507	DQ417222	S20K,S23F,F25L,F26C,I27V,F28C,L29F,F30L,A34T,T35A,A37G,E38A,S39A,D49E,V67F,P68S,A78T,E79D,K90T,T97N,S112T,R132Q,T170K,N177S,P198Q,R208K,I223V,Y227D,I241L,L250I,K252M,P261S,V266I,L302R,L304W,K319Q
110350569	AM283490	R102P,V202L

295002614	GU944726	S23F,I27M,A34T,T35A,A37G,V67I,A78T,E79D,K94N,T97N,K108R,R132Q,T170K,N171Y,N177S,N183K,P194S,T197A,R208N,K215R,I216V,I223V,I241L,K252M,G254bS,S262G,E265D,V266I,A297S,L304W,A307T,L311F,P317S,S318T,K319T,P320A,S321H,N322-
182382568	EU588392	S23F,I27M,A34T,T35A,A37G,V67I,A78T,E79D,K94N,T97N,K108R,R132Q,T170K,N171Y,N177S,N183K,T197A,R208N,K215R,I216V,I223V,I241L,K252M,G254bS,S262G,E265D,V266I,A297S,L304W,A307T,L311F,P317S,S318T,K319T,P320A,S321H,N322-
217038357	ACJ76644	N77D,R132Q,T170K,S174G,V201L,I241L,K252I,V266A,P320L
40644311	CAD55935	S20K,S23F,F25L,F26C,I27V,F28C,L29F,A34T,T35A,A37G,E38A,S39A,D49E,Y53F,P68T,A78T,E79D,K90T,T97N,K108E,S112T,R132Q,D149S,T170K,N171Y,N177S,P198Q,V201L,R208S,K215E,Y227H,I241L,L250I,K252M,P261S,V266K,L300M,L302R,L304W,V308L,N312K,P317T,K319S

Table S2. Mutations shared by all variants of the respective network in comparison to the reference sequence IMP-1

**Network**	Mutations
**E-1**	S20K,S23F,F25L,F26C,A34T,T35A,A37G,E38A,D49E,P68S,A78T,E79D,T97N,R132Q,E140V,T170K,N177S,P198Q,E207K,R208N,I223V,G235D,I241V,K246H,K249E,L250K,K252I,K255N,E265D,V266I,L304W,P317S,S318N,K319T,P320V,S321H,N322-
**E-2**	S23F,I27M,A34T,T35A,A37G,V67I,A78T,E79D,K94N,T97N,K108R,R132Q,T170K,N171Y,N177S,N183K,T197A,R208N,K215R,I216V,I223V,I241L,K252M,G254bS,E265D,V266I,A297S,L304W,A307T,L311F,P317S,S318T,K319T,P320A,S321H,N322-
**D**	S20K,S23F,F25L,F26C,I27V,F28C,L29F,F30L,A34T,T35A,A37G,E38A,S39A,D49E,P68S,A78T,E79D,K90T,T97N,S112T,R132Q,T170K,N177S,P198Q,R208K,I223V,Y227D,I241L,L250I,K252M,G254bVP261S,V266I,L302R,L304W,K319Q
**C**	S20K,S23F,F25L,F26C,I27V,F28C,L29F,F30L,A34T,T35A,A37G,E38A,S39A,D49E,P68S,A78T,E79D,K90T,T97N,S112T,R132Q,T170K,N177S,P198Q,R208K,I223V,Y227D,I241L,L250I,K252M,P261S,V266I,L302R,L304W,K319Q
**B-1**	S39P, N77D,R132Q,T170K,S174G,V201L,I241L,K252I, V266A,P320L
**B-2**	N77D,R132Q,T170K,S174G,V201L,I241L,K252I, V266A,P320L

Table S3. Triplets of IMP variants with predicted missing variant to complement the triplet to a quartet.

Network	Triplet variants	Mutations relative to network	Predicted variant in respective network
A	IMP-1 – IMP-10 – IMP-40	V67F – V67F + F87S	F87S
	IMP-1 – IMP-10 – IMP-30	V67F – E59K	V67F + E59K
	IMP-1 – IMP-10 – IMP-34	V67F – E126G	V67F + E126G
	IMP-1 – IMP-10 – IMP-42	V67F – G63R	V67F + G63R
	IMP-1 – IMP-10 – IMP-6	V67F – S262G	V67F + S262G
A	IMP-1 – IMP-6 – IMP-25	S262G – S262G + G235S	G235S
	IMP-1 – IMP-6 – IMP-30	S262G – E59K	S262G + E59K
	IMP-1 – IMP-6 – IMP-42	S262G – G63R	S262G + G63R
A	IMP-1 – IMP-30 – IMP-34	E59K – E126G	E59K + E126G
	IMP-1 – IMP-30 – IMP-42	E59K – G63R	E59K + G63R
A	IMP-6 – IMP-3 – IMP-25	S262G – E126G + S262G – S262G + G235S	E126G + S262G + G265S
B-1	IMP-4 – IMP-26 – IMP-38	V67F – S262G	V67F + S262G
C	IMP-9 – s182 – s295	S262G – S262G + P194S	P194S
E-1	IMP-19 – IMP-2 – IMP-20	V67F – K315R	V67F + K315R
E-2	IMP-8 – IMP-24 – s90	V67F – S39R	V67F + S39R

Table S4. Pairs of IMP variants which differ by 2 mutations with no known intermediary variant. Intermediary variants for possible pathways were predicted.

Network	Pair variants	Mutations relative to network	Predicted variants
A	IMP-1 – 110350569	R102P + V202L	R102P and V202L

Table S5. Predicted S262G and V67F variants

Network	Expected variants relative to network
B1	V67F, S262G
C	V67F
D	S262G
E-1	S262G
E-2	S262G

Figures

	wild type	mutant 1
Mutant 1	1	-
Mutant 2	1	2

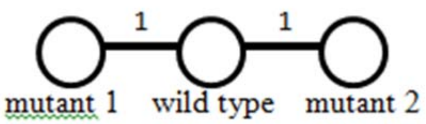

Figure S1. The distance matrix and a network representation of a triplet of sequences consisting of a wild type and two single mutants thereof (mutant 1 and mutant 2)

	wild type	mutant 1	mutant 2
Mutant 1	1	-	
Mutant 2	1	2	-
Double mutant	2	1	1

Figure S2. The distance matrix of a quartet of sequences consisting of a wild type, two single mutants thereof (mutants1 and 2), and the respective double mutant

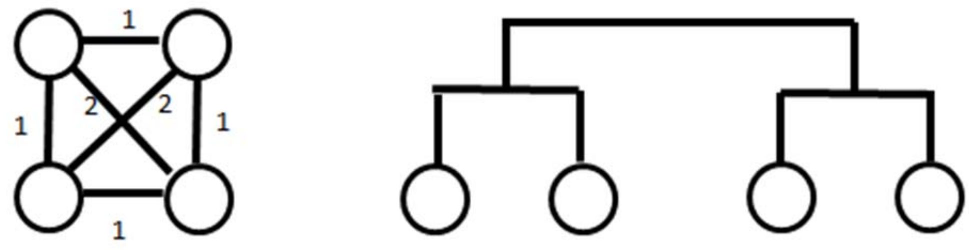

Figure S3 Left: Network representation of a quartet of sequences consisting of a wild type, two single mutants thereof (mutants1 and 2), and the respective double mutant (derived from the distance matrix in Figure S2). Right: the four sequences cannot be represented by a binary tree assuming additivity of distances

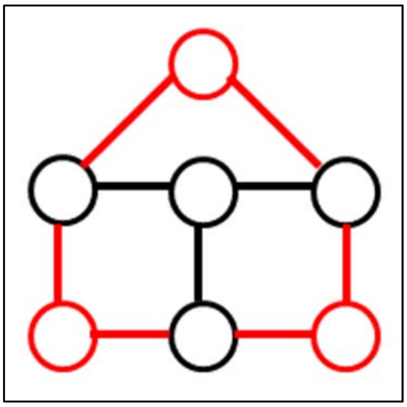

Figure S4 From a network of four variants of known sequence (black), three variants (red) can be predicted that are expected to be functional and to exist in the biosphere

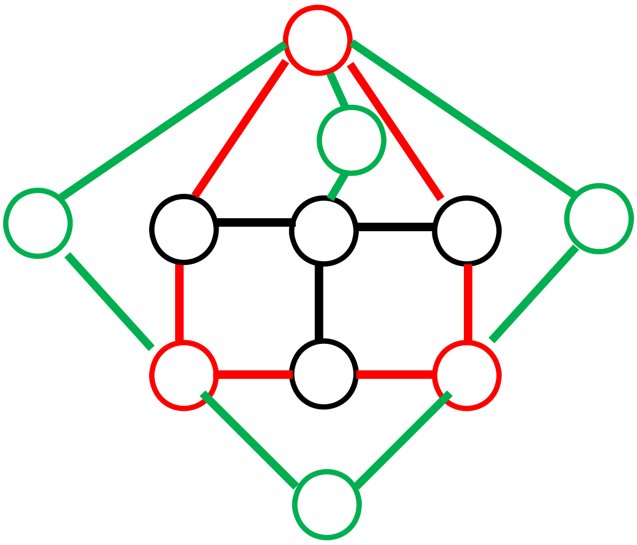

Figure S5 From the network constructed in Figure S4, four further mutants (green) can be predicted that are expected to be functional and to exist in the biosphere
